# METTL3 promotes lung adenocarcinoma tumor growth and inhibits ferroptosis by stabilizing SLC7A11 m^6^A modification

**DOI:** 10.1186/s12935-021-02433-6

**Published:** 2022-01-07

**Authors:** Yiming Xu, Dandan Lv, Chao Yan, Hua Su, Xue Zhang, Yangfeng Shi, Kejing Ying

**Affiliations:** 1 Department of Respiratory and Critical Medicine, Sir Run Run Shaw Hospital, Zhejiang University School of Medicine, No. 3, Qingchun East Road, Hangzhou, China; 2 Cancer Center, Zhejiang University, Hangzhou, China; 3 Department of Pathology and Pathophysiology, Zhejiang University School of Medicine, Hangzhou, China; 4 Department of Respiratory and Critical Medicine, Affiliated Hangzhou First People’s Hospital, Zhejiang University School of Medicine, No.261, Huansha Road, Hangzhou, China

**Keywords:** Lung adenocarcinoma, METTL3, N6-methyladenosine (m^6^a) modification, Ferroptosis, SLC7A11

## Abstract

**Background:**

N6-methyladenosine (m^6^A) has emerged as a significant regulator of the progress of various cancers. However, its role in lung adenocarcinoma (LUAD) remains unclear. Here, we explored the biological function and underlying mechanism of methyltransferase-like 3 (METTL3), the main catalyst of m^6^A, in LUAD progression.

**Methods:**

The expression of m^6^A, METTL3, YTHDF1 and SLC7A11 were detected by immunochemistry or/and online datasets in LUAD patients. The effects of METTL3 on LUAD cell proliferation, apoptosis and ferroptosis were assessed through in vitro loss-and gain-of-function experiments. The in vivo effect on tumorigenesis of METTL3 was evaluated using the LUAD cell xenograft mouse model. MeRIP-seq, RNA immunoprecipitation and RNA stability assay were conducted to explore the molecular mechanism of METTL3 in LUAD.

**Results:**

The results showed that the m^6^A level, as well as the methylase METTL3 were both significantly elevated in LUAD patients and lung cancer cells. Functionally, we found that METTL3 could promote proliferation and inhibit ferroptosis in different LUAD cell models, while METTL3 knockdown suppressed LUAD growth in cell-derived xenografts. Mechanistically, solute carrier 7A11 (SLC7A11), the subunit of system Xc^−^, was identified as the direct target of METTL3 by mRNA-seq and MeRIP-seq. METTL3-mediated m^6^A modification could stabilize SLC7A11 mRNA and promote its translation, thus promoting LUAD cell proliferation and inhibiting cell ferroptosis, a novel form of programmed cell death. Additionally, we demonstrated that YTHDF1, a m^6^A reader, was recruited by METTL3 to enhance SLC7A11 m^6^A modification. Moreover, the expression of YTHDF1 and SLC7A11 were positively correlated with METTL3 and m^6^A in LUAD tissues.

**Conclusions:**

These findings reinforced the oncogenic role of METTL3 in LUAD progression and revealed its underlying correlation with cancer cell ferroptosis; these findings also indicate that METTL3 is a promising novel target in LUAD diagnosis and therapy.

**Supplementary Information:**

The online version contains supplementary material available at 10.1186/s12935-021-02433-6.

## Background

Despite advances in cancer progression and treatment, lung cancer remains occupying the leading cause of cancer-related mortalities worldwide, with an estimated 2.2 million new cases and 1.8 million deaths per year [1, 2]. Approximately 85% of patients are diagnosed with non-small-cell lung cancer (NSCLC), of which lung adenocarcinoma (LUAD) is the most frequent histological manifestation [3]. Until now, the overall survival time of LUAD patients remains poor, despite the advancement of surgical resection, chemotherapy, radiotherapy, molecular targeted therapy and immunotherapy. Therefore, further mechanism exploration is necessary for improving the diagnosis and prognosis of LUAD at the outset.

N6-methyladenosine (m^6^A), one of the universal modifications of RNA molecules among numerous epigenetic changes, drives multiple biological functions, including tissue development, stemness maintenance and differentiation, DNA damage response and metabolism [4–7]. Alteration of m^6^A participates in regulating mRNA splicing, export, translation, and stability, which involves three components, including methyltransferases (writers), demethylases (erasers) and RNA-binding proteins (readers) [8]. The m^6^A methyltransferase complex, contains a core subunit, methyltransferase-like 3 (METTL3), and other accessory regulators including METTL14, Wilms’ tumor 1-associating protein (WTAP), VIRMA, RBM15 and ZC3H13, which catalyzes the m^6^A modification, while demethylases, including fat mass and obesity-associated (FTO) and AlkB homolog 5 (ALKBH5), reverse this effect. Additionally, the YT521-B homology (YTH) domain family of proteins (YTHDF1/2/3 and YTHDC1/2), and insulin-like growth factor 2 mRNA binding proteins (IGF2BP1/2/3) are recognized as RNA-binding proteins that bind m^6^A sites and lead target RNA to different destination [9]. Recently, m^6^A and its associated proteins were reported to be essentially responsible for tumorigenesis and cancer progression in various cancer types, including lung cancer [9–11]. For example, METTL3 was found essential for TGF-β-induced epithelial-mesenchymal transition of lung cancer cells [12]; YTHDF2 could promote lung cancer cell growth via facilitating 6PGD mRNA translation [13]. Yet the biological significance and underlying mechanism of m^6^A in LUAD remain elusive.

Ferroptosis, the new-found cell death form characterized by iron accumulation and lipid-reactive oxygen species (ROS) within the cell, is distinct both morphologically and functionally from other known forms of cell death including necrosis, apoptosis and autophagy [14, 15]. Increasing evidences have demonstrated that ferroptosis is a crucial regulator of cancer progression and may be harnessed in cancer therapies [16, 17]. Liu et al. [18] found that nuclear factor-erythroid 2-like 2 (NRF2) inhibitor (brusatol) could enhance the sensitivity of NSCLC cells to cystine deprivation-induced ferroptosis depending on FOCAD-FAK signaling, and the combination of brusatol and erastin showed better therapeutic effect of NSCLC. Chen et al. [19] showed erainin, a novel dibenzyl compound, could induce ferroptotic cell death in lung cancer cells by Ca^2+^/CaM-dependent pathway. All of these suggest the potential anti-tumor effect of ferroptosis inducers in LUAD treatment in further researches. Moreover, several studies gradually revealed the potential connections between m^6^A and ferroptosis. For example, YTHDF1 was found to be closely related to iron metabolism and tumor progression in hypopharyngeal squamous cell carcinoma by targeting TFRC via m^6^A-dependent mechanism [20]. Exosomal miR-4443 could promote cell resistance to cisplatin in NSCLC via FSP1 m^6^A-mediated ferroptosis [21]. Nevertheless, the relationship between m^6^A and ferroptosis in LUAD still need further exploration.

In this study, we found that the level of m^6^A and the expression of METTL3 were increased in LUAD patients. Then, we demonstrated that the METTL3-mediated m^6^A modification in LUAD could promote tumorigenesis and inhibit cell ferroptosis via SLC7A11 regulation. Moreover, the METTL3-mediated methylation of SLC7A11 could regulate the stability and translation of SLC7A11 mRNA through YTHDF1 recruitment in LUAD cells. We also found that both the expression of YTHDF1 and SLC7A11 were elevated and positively related to METTL3 and m^6^A levels in LUAD samples, which indicated that METTL3-mediated m^6^A modification of SLC7A11 illustrated a significant role in human LUAD progression and ferroptosis pathways.

## Materials and methods

### Patient samples

All LUAD tissues and paired adjacent normal lung tissues were obtained from LUAD patients who underwent surgery at Sir Run Run Shaw Hospital from January 2019 to January 2020 without previous chemotherapy or radiotherapy. Written informed consent was obtained from each patient in this study, and protocols were approved by the ethical committee of Sir Run Run Shaw Hospital. All specimens were immediately frozen in liquid nitrogen after removal. And all samples were examined by experienced pathologists who confirmed the disease diagnosis.

### Cell culture and reagents LC

NSCLC cell lines (NCl-H1975, A549, PC9, NCl-H322, NCl-H460, SPC-A1, NCl-H1299), human normal lung epithelial cells (BEAS-2B) were obtained from American Type Culture Collection (ATCC). NSCLC cell lines and BEAS-2B cells were cultured in RPMI-1640 medium (Solarbio, China), supplemented with 10% fetal bovine serum (FBS, Noverse), 100 units/mL penicillin and 100 μg/mL streptomycin (Solarbio) in an incubator with 5% CO_2_ at 37 °C. All human cell lines have been authenticated using short tandem repeat profiling within the last 3 years. All experiments were performed with mycoplasma-free cells.

### siRNA, shRNA and plasmid constructs

The siRNAs for METTL3 and YTHDF1, and lentivirus for METTL3 knockdown were synthesized by GenePharma (Shanghai, China). The sequences were as follows: siMETTL3#1 (sense: 5′-GCUACCUGGACGUCAGUAUTT-3′, antisense: 5′-AUACUGACGUCCAGGUAGCTT-3′); siMETTL3#2 (sense: 5′-GGUUGGUGUCAAAGGAAAUTT-3′, antisense: 5′-AUUUCCUUUGACACCAACCTT-3′); siYTHDF1 (sense: 5′-GGAGAAUAACGACAACAAATT-3′, antisense: 5′-UUUGUUGUCGUUAUUCUCCTT-3′); and shMETTL3 (5′-GCAAGAATTCTGTGACTATGG-3′).

The pEX-3-METTL3 expression plasmid was synthesized by GenePharma (Shanghai, China). The pcDNA3.1-SLC7A11 expression plasmid was synthesized by Genomeditech (Shanghai, China).

### Cell transfection and lentiviral infection

For transient transfection, siRNAs and plasmids were transfected into cells using Lipofectamine 3000 (Invitrogen, L3000015) according to the manufacturer’s protocol. The medium was refreshed 4–6 h after transfection. Cells were applied for other assays after 24 to 48 h’ transfection.

For stable transfection, the lentivirus packaged with short hairpin RNA (shRNA) was transduced into cells using polybrene (5 μg/mL). After 48 h, stably transfected cells were selected with puromycin (1 μg/mL) for 2–7 days.

### RNA isolation and real-time quantitative PCR (RT-qPCR)

Total RNA of the indicated cells was extracted according to the manufacturer’s protocol using RNA extract reagent (Axygen, AP-MN-MS-RNA-250). The purity and concentration of RNA were determined by measuring the absorbance at A260/280 nm using a Nanodrop 2000 (Thermo Fisher Scientific). Reverse transcription of RNA (1 μg) was performed prior to cDNA amplification using a HiFiScript cDNA Synthesis Kit (CWBIO, CW2569). Then, RT-qPCR was performed in a QuantStudio (Applied Biosystem, ABI) system using SYBR Premix Ex Taq™ II (Takara, RR820A) with primers. β-actin served as an endogenous control to quantify the relative expression of targeted genes using the 2^−ΔΔCt^ method. The primers synthesized by TSINGKE were listed in Additional file 2: Table S1.

### Western blot and antibodies

Total protein was extracted using RIPA lysis buffer and was quantified using a BCA protein assay kit (Beyotime, China). Equal amounts of proteins were separated by 10% sodium dodecyl sulfate–polyacrylamide gel electrophoresis (SDS-PAGE) and then transferred onto polyvinylidene fluoride (PVDF) membranes (Bio-Rad, USA). The membranes were blocked with 5% nonfat milk for 1 h at room temperature and then incubated with primary antibodies at 4 °C overnight. After three washes the following day, the membranes were incubated with HRP-conjugated secondary antibodies (CST, #7076, #7074, 1:4000 dilution) for 2 h at room temperature. An ECL detection system (FDbio) was used for visualization. β-actin or α-tubulin served as internal controls. The primary antibodies used in this study were as follows: β-actin (CST, #8457, 1:1000), α-tubulin (Sigma, T6199, 1:5000), METTL3 (Abcam, ab195352, 1:1000), SLC7A11 (CST, #12691, 1:1000), and YTHDF1 (Proteintech, 17479–1–AP, 1:1000).

### Immunohistochemistry (IHC) and TUNEL assay

Tissues were fixed in 4% paraformaldehyde and embedded in paraffin. All slides containing tissue sections were incubated with the indicated primary antibodies (m^6^A, Synaptic Systems, 202003, 1:100; METTL3, Abcam, ab195352, 1:500; Ki67, Abcam, ab92742, 1:1000; YTHDF1, Proteintech, 17479–1–AP, 1:200; SLC7A11, Proteintech, 26864–1–AP, 1:200) in a humidified chamber at 4 °C overnight. The Immunohistochemical staining was visualized with diaminobenzidine, with a hematoxylin counterstain to observe nuclei. Protein expression was assessed according to the intensity (1, 0–25%; 2, 26–50%; 3, 51–75%; 4, 76–100%) and extent of staining (0, negative; 1, weak; 2, moderate; 3, strong) under microscopy. IHC scores were obtained by multiplying the intensity by the extent of staining, and the scores of 0–6 and of 8–12 were classified as low and high expression respectively.

The TUNEL assay was performed according to the instruction of The One Step TUNEL Apoptosis Assay Kit (Beyotime, C1089), and cell nuclei were co-stained with Hoechest 33342.

### Cell proliferation assays

For the CCK-8 assay, cells were seeded in 96-well plates at a density of 5000 cells per well one day before transfection or ferrostatin-1 (Fer-1, 1 μM) treatment. Then, each well was administered with CCK-8 solution from a kit (APExBIO, USA, K1018) at the indicated time points (0, 1, 2, 3 days after treatment). The absorbance at 450 nm was then measured.

The EdU assay was performed using the BeyoClick™ EdU-555 cell proliferation kit (Beyotime, C0075S) according to the manufacturer’s instructions. Cells were seeded in 24-well plates at a density of 5 × 10^4^ cells per well one day before treatment.

### Cell cycle and apoptosis assays

Cell cycle and apoptosis assays were analyzed by flow cytometry analysis using FACS (BD Biosciences). For the cell cycle analysis, cells were harvested by trypsinization and then fixed in ice-cold 75% ethanol at − 20 °C overnight. The next day, the cells were stained with propidium iodide (PI) according to the Cell Cycle Staining Kit (MultiSciences, China, CCS012). Cell cycle distributions were determined by ModFitLT Software. For the cell apoptosis assay, cells were carefully harvested by trypsinization and stained using an Annexin V-FITC/PI Cell Apoptosis Kit (Beyotime, C1062). The percentage of apoptotic cells was analyzed by FlowJo software.

### Intracellular ROS and malondialdehyde (MDA) measurement

Intracellular ROS levels were detected by the peroxide-sensitive fluorescent probe 2’, 7’-dichlorofluorescein diacetate (DCFH-DA, Sigma-Aldrich, D6883). Briefly, after the indicated treatment, cells in 6-well plates were washed with PBS and incubated with 10 μM DCFH-DA for 30 min under standard conditions. Cells were then washed and collected, and the mean fluorescence intensity of DCFH-DA, which was representative of ROS level, was measured by flow cytometry. The results were analyzed using FlowJo software.

For intracellular MDA measurement, lipid Peroxidation MDA assay kit (Beyotime, S0131S) was used according to the manufacturer’s instructions. The supernatants reacted with thiobarbituric acid (TBA) in each sample, and the levels of MDA were finally evaluated by measuring the absorbance at 532 nm. Then, the MDA levels were normalized to the cell protein contents as nmol/mg protein.

### Quantification of total m^6^A RNA

The m^6^A content of 200 ng RNA extracted from the indicated cells was analyzed using the EpiQuik m^6^A RNA Methylation Quantification Kit (Colorimetric) (Epigentek, USA, P-9005–48) following the manufacturer’s instructions. The m^6^A level was quantified by measuring the absorbance of each well at 450 nm, and the standard curve was then used to calculate the m^6^A level.

### Methylated RNA immune-precipitation (MeRIP)-seq

As previously reported [20, 22], total RNA was extracted using TRIzol reagent (Ambion, USA, 223408). The total RNA quality and quantity were analysis of Bioanalyzer 2100 and RNA 6000 Nano LabChip Kit (Agilent, CA, USA) with RIN number > 7.0. Approximately 50 μg of total RNA was subjected to isolation of poly (A) mRNA with poly-T oligo-attached magnetic beads (Invitrogen). The cleaved RNA fragments were incubated for 2 h at 4 °C with an m^6^A-specific antibody (Synaptic Systems, Germany, No. 202003) in IP buffer (50 mM Tris–HCl, 750 mM NaCl and 0.5% Igepal CA-630) supplemented with BSA. The mixture was then incubated with protein-A beads and eluted with elution buffer (1 × IP buffer and 6.7 mM m^6^A). Eluted RNA was precipitated by 75% ethanol. Eluted m^6^A-containing fragments (IP) and untreated input control fragments are converted to the final cDNA library in accordance with a strand-specific library preparation by the dUTP method. Finally, we performed 2 × 150 bp paired-end sequencing on an Illumina NovaSeq™ 6000 platform at LC-BIO Bio-tech ltd (Hangzhou, China) according to the vendor’s recommended protocol.

### RNA immune-precipitation (RIP)

The RIP assay was performed using a Magna RIP™ RNA-Binding Protein Immunoprecipitation Kit (Millipore, USA, 17–700) according to the manufacturer’s instructions. Briefly, the indicated cell lysates were collected and incubated with magnetic bead protein A/G (CS203178) coated with 5 μg of control IgG antibody (PP64B), anti-m^6^A antibody (Synaptic Systems, 202003), or anti-YTHDF1 antibody (Proteintech, 17479-1-AP) with rotation at 4 °C overnight. The next day, RNA was purified and extracted using the phenol: chloroform: isoamyl alcohol method. The relative expression of SLC7A11 was detected by RT-qPCR. IP enrichment was normalized to the input yielded from the same number of cells.

### RNA stability assay

RNA stability assays were performed as previously described [23]. Briefly, cells were treated with actinomycin D (APExBIO, A4448) for 0 h, 3 h, or 6 h at a final concentration of 5 μg/mL. Then, total RNA was extracted for RT-qPCR to quantify the relative expression of SLC7A11 mRNA. The degradation rate of mRNA (*K*_decay_) was calculated using the following equation: ln(C/C_0_) = − *K*_decay_t. The half-life (t_1/2_) of mRNA was calculated using the equation: In(1/2) = − *K*_decay_t_1/2_.

### Animal experiment

Female BALB/c-nude mice (5 weeks of age) were used for xenografts models and raised under specific pathogen-free conditions. The animal experiments were approved by The Institutional Animal Care and Use Committee of Zhejiang University. For the subcutaneous xenograft model, PC9 cells stably transfected with METTL3 knockdown (shMETTL3) or negative control (shNC) shRNA (5 × 10^6^ cells per mouse, n = 6) were suspended in 200 μl PBS with 50% Matrigel matrix (Corning, USA, 354234) and then injected into one side of the axilla of nude mice. Tumor growth and volume were measured every 3 days, and the tumor volume was assessed using the formula: volume (mm^3^) = longer diameter × shorter diameter^2^/2. After 3 weeks, the mice were sacrificed with 2% pentobarbital sodium (100 mg/kg) and tumor weight was determined.

### Statistical analysis

Data between two groups were analyzed using two-tailed unpaired Student’s t-test while one-way or two-way ANOVA was used for multiple comparisons using GraphPad Prism 8.0 and SPSS 20.0. The data are showed as the mean ± standard deviation (SD) from at least three independent experiments. A P-value less than 0.05 was considered statistically significant.

## Results

### METTL3-mediated m^6^A modification is elevated in LUAD patients and NSCLC cells

**Fig. 1:**
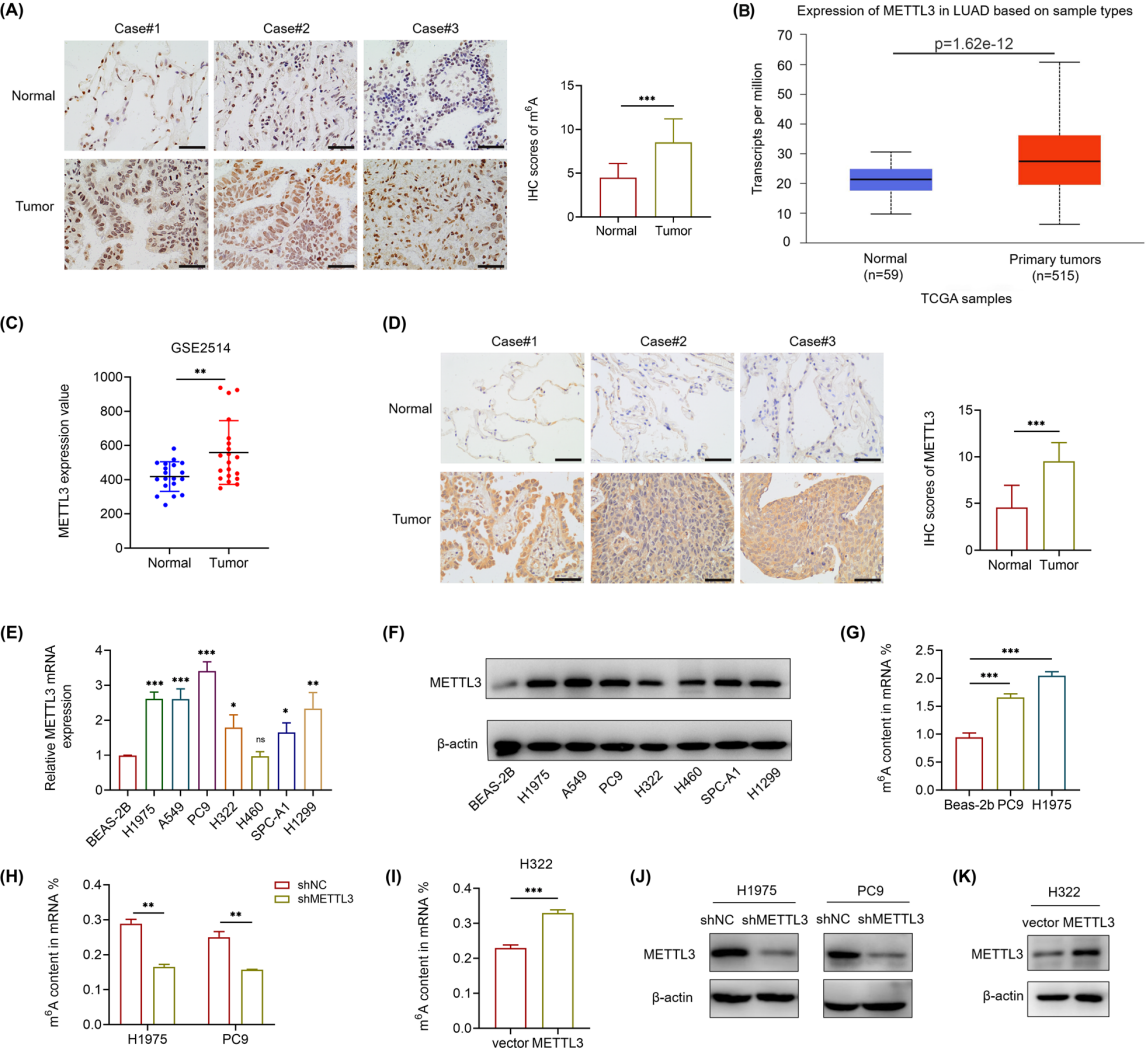
HYPERLINK "sps:id::fig1||locator::gr1||MediaObject::0" METTL3 regulates m^6^A level in LUAD patients and NSCLC cells. **A** Representative IHC images and IHC scores of m^6^A level in 21 LUAD tissues and adjacent normal tissues (Scale bar: 50 μm). **B** Expression of METTL3 in LUAD patients based on TCGA samples using UALCAN platform. **C** Expression of METTL3 in LUAD patients based on GEO datasets (GSE2514). **D** Representative IHC images and IHC scores of METTL3 in 21 LUAD tissues and adjacent normal tissues (Scale bar: 50 μm). **E** and **F** Relative mRNA levels measured by RT-qPCR and protein levels analyzed by western blot of METTL3 in NSCLC cell lines (H1975, A549, PC9, H322, H460, SPC-A1 and H1299), compared with normal human lung epithelia cells (BEAS-2B). **G** Quantitative analysis of the percentage of m^6^A content in LUAD cells (H1975 and PC9), compared with BEAS-2B cells. **H** Quantitative analysis of the percentage of m^6^A content in METTL3 stable knockdown H1975 and PC9 cells. **I** Quantitative analysis of the percentage of m^6^A content in METTL3 overexpression H322 cells. **J** Transfection efficiencies of METTL3 stable knockdown in H1975 and PC9 cells were confirmed by western blot. **K** The overexpression efficiency of METTL3 after 24 h transfection of METTL3 or vector plasmid in H322 cells. *P < 0.05, **P < 0.01, ***P < 0.001, ns, not significant

To investigate the role of m^6^A modification in LUAD, we first evaluated the m^6^A level in LUAD tissues by immunohistochemistry (IHC). The m^6^A level was increased compared with adjacent normal tissues (Fig. 1A). Since m^6^A writers are the main catalysts that lead m^6^A modifications^9^, we analyzed different writers, including METTL3, METTL14, WTAP, ZC3H13 and RBM15, in The Cancer Genome Atlas (TCGA) database using the UALCAN platform [24]. METTL3 was the most significantly elevated gene in LUAD samples (Fig. 1B, Additional file 1: Fig. S1A–D). In addition, the elevated expression of METTL3 was also confirmed in the Gene Expression Omnibus (GEO) database (GSE2514) (Fig. 1C) and our LUAD tissues by IHC (Fig. 1D). In the NSCLC cell lines, RT-qPCR and western blot also indicated that both the mRNA and protein levels of METTL3 were elevated compared with those in BEAS-2B cells, which are normal human lung epithelial cells (Fig. 1E, F). Consistently, the mRNA content of m^6^A in LUAD cells was much higher than that in BEAS-2B cells, as measured by m^6^A quantitative measurement (Fig. 1G). Furthermore, knockdown of METTL3 noticeably decreased the m^6^A level in LUAD cells, while METTL3 overexpression had the opposite effect (Fig. 1H, I). The transfection efficiencies were confirmed by western blot (Fig. 1J, K). These results collectively reveal that METTL3 regulate the m^6^A level in LUAD patients and NSCLC cells.

### METTL3 promotes LUAD proliferation and inhibits apoptosis in vitro and in vivo

**Fig. 2:**
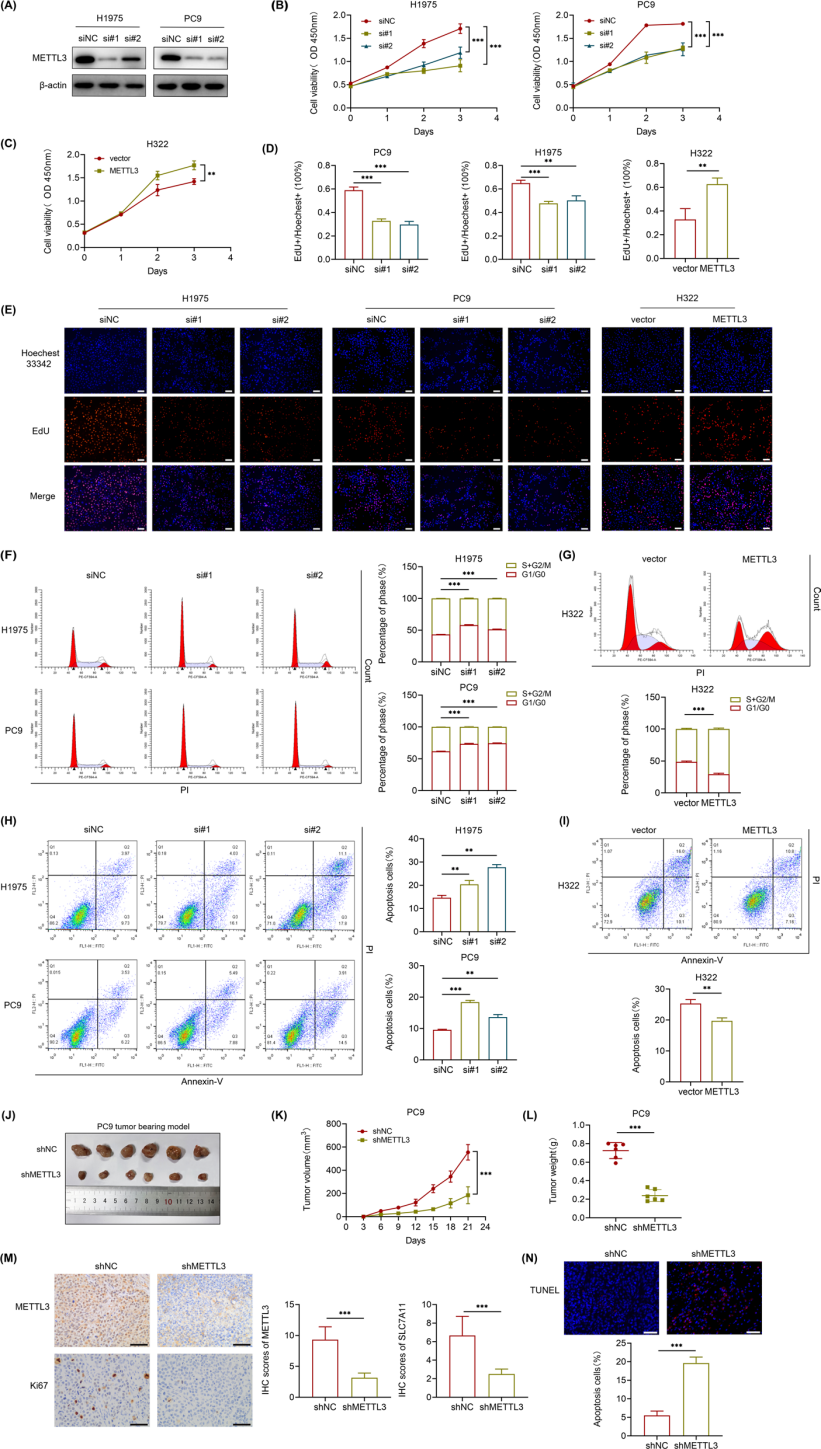
METTL3 promotes LUAD proliferation and inhibits cell apoptosis. **A** The knockdown efficiency of METTL3 after 48 h transfection of METTL3 siRNA (si#1, si#2) and negative control siRNA (siNC) in H1975 and PC9 cells were confirmed by western blot. **B** and **C** CCK-8 assays showed the growth curves of LUAD cells upon METTL3 knockdown in H1975 and PC9 cells, and METTL3 overexpression in H322 cells respectively. **D** and **E** Representative images of EdU staining and the ratio of EdU positive cells to total Hoechest 33,342 positive cells (Scale bar: 100 μm). **F** and **G** Flow cytometry analysis for cell cycle of cellular distribution by PI staining with METTL3 knockdown in H1975 and PC9 cells, and METTL3 overexpression in H322 cells. **H** and **I** Flow cytometry analysis for apoptotic cell proportion (Q2 + Q3) by Annexin V-FITC/PI staining. **J** Photograph of dissected subcutaneous tumors at sacrificed time. **K** The tumor growth curves of subcutaneous xenograft models with stable METTL3 knockdown (shMETTL3) and negative control (shNC) PC9 cells injection (n = 6). **L** Tumor weight of dissected subcutaneous tumors at sacrificed time. **M** Representative IHC images and IHC scores of METTL3 and Ki67 stained in xenograft tumors (Scale bar: 50 μm). **N** Representative TUNEL images and apoptotic cell proportion of xenograft tumors (Scale bar: 50 μm). **P < 0.01, ***P < 0.001

To explore the correlation of METTL3 level and the malignancy of LUAD, we also knocked down METTL3 in PC9 and H1975 cells using two specific small interferon RNAs (siRNAs, named si#1 and si#2). The efficiency of knockdown was confirmed by western blot (Fig. 2A). LUAD cell growth decreased remarkably upon METTL3 knockdown, as determined by CCK-8 assays, while overexpression increased cell growth (Fig. 2B, C). Furthermore, EdU staining demonstrated that METTL3 knockdown inhibited cell proliferation, in contrast, overexpression substantially promoted cell proliferation (Fig. 2D, E). Since METTL3 distinctly regulated LUAD cell proliferation, we then assessed its impact on the cell cycle. As substantiated by flow cytometry analysis, the cell cycle was arrested in G0/G1 phase and the number of cells in S/G2 phase was reduced due to METTL3 knockdown (Fig. 2F); consistently, METTL3 overexpression resulted in the opposite effect (Fig. 2G). In addition, the proportion of apoptotic cells was increased when METTL3 was knocked down, while overexpression decreased it (Fig. 2H, I). To further evaluate the oncogenic role of METTL3 in LUAD in vivo, we applied subcutaneous xenograft models established with stable METTL3 knockdown (shMETTL3) PC9 cells and control (shNC) PC9 cells. Consistent with the in vitro results, the tumors of the METTL3-deficient group grew more slowly than those of the control group (Fig. 2J, K). Moreover, the average tumor volume and tumor weight at killing were both remarkably decreased in the shMETTL3 group compared with the shNC group (Fig. 2L). Then, we evaluated the cell proliferation marker Ki-67 by IHC and the proportion of apoptotic cells by TUNEL assay in these solid tumors. As expected, the IHC score for Ki-67 in shMETTL3 group was significantly reduced while the proportion of apoptotic cells was increased compared with those in the control group (Fig. 2M, N). Overall, these loss-and gain-of-function assays confirm that METTL3 promotes LUAD proliferation and inhibits apoptosis both in vitro and in vivo.

### SLC7A11 is a target of METTL3 in LUAD depending on its m^6^A methyltransferase activity

**Fig. 3:**
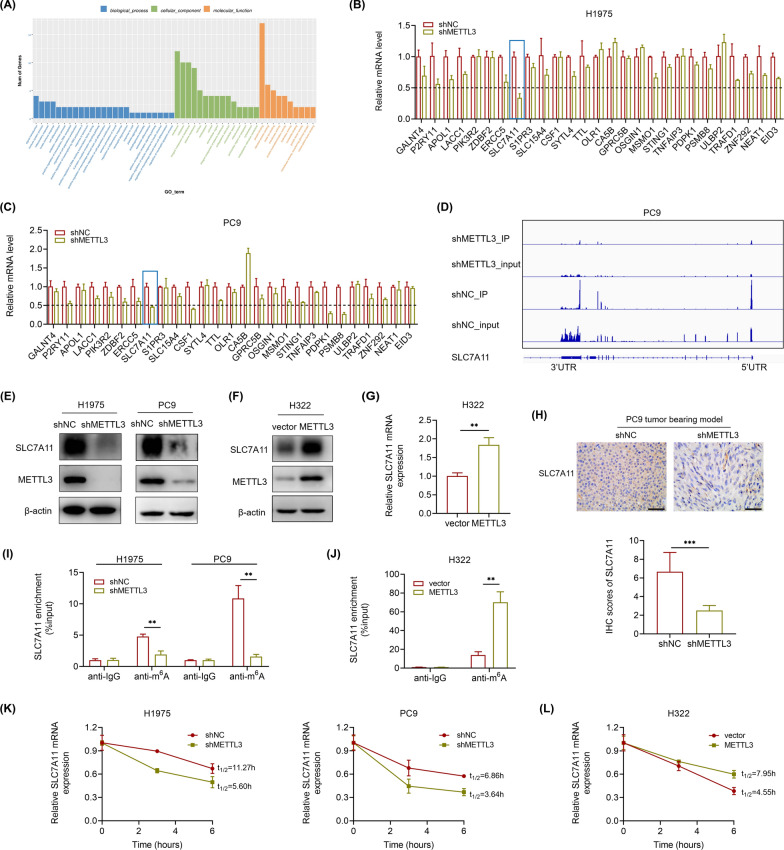
SLC7A11 acts as a target of METTL3 depending on its m^6^A methyltransferase activity. **A** GO analysis of MeRIP-seq data. **B** and **C** Relative mRNA levels measured by RT-qPCR of selected genes in METTL3 knockdown H1975 and PC9 cells. **D** The relative abundance of m^6^A peaks of SLC7A11 mRNA in METTL3 knockdown and control PC9 cells analyzed by MeRIP-seq. **E** The protein levels showed by western blot of SLC7A11 with METTL3 knockdown in H1975 and PC9 cells. **F** and **G** The expression level of SLC7A11 mRNA and protein showed by RT-qPCR and western blot in METTL3 overexpression H322 cells. **H** Representative IHC images and IHC scores of SLC7A11 stained in xenograft tumors (Scale bar: 50 μm). **I** and **J** The m^6^A levels of SLC7A11 mRNA measured by MeRIP-qPCR analysis after METTL3 knockdown or METTL3 overexpression. **K** and **L** SLC7A11 mRNA half-lives (t_1/2_) showed by RNA decay rates followed by RT-qPCR after METTL3 knockdown in H1975 and PC9 cells and METTL3 overexpression in H322 cells. Data were collected at indicated timepoints (0 h, 3 h, and 6 h) with actinomycin D (Act D, 5 μg/mL) treatment. **P < 0.01, ***P < 0.001

To determine whether the mechanism of the oncogenic role of METTL3 in LUAD depends on its m^6^A methyltransferase activity, RNA sequencing (RNA-seq) and m^6^A-modified RNA immunoprecipitation sequencing (MeRIP-seq) were performed in PC9 cells with stable METTL3 knockdown and control PC9 cells. GO analysis of MeRIP-seq revealed that METTL3 and METTL3-mediated m^6^A methylation participate in multiple biological processes and molecular functions as well as cellular components (Fig. 3A). Then, we further screened genes with hypomethylated m^6^A with decreased expression in METTL3 knockdown cells (p < 0.05) and filtered them according to downregulated genes in the RNA-seq analysis (p < 0.05). There were 27 genes chosen, and in our verification qPCR analysis, SLC7A11 (also known as xCT) was found to be the only gene that down expressed consistently (fold change < 0.5) in H1975 and PC9 cells when METTL3 was knocked down (Fig. 3B, C). Besides, the m^6^A peaks of SLC7A11 were remarkably reduced in METTL3 knockdown PC9 cells compared with control cells (Fig. 3D). Thus, SLC7A11 was selected as a further candidate target of METTL3 in LUAD. In our validation assays, the protein levels of SLC7A11 were also found significantly decreased after METTL3 knockdown in H1975 and PC9 cells (Fig. 3E). Conversely, overexpression of METTL3 in H322 cells indeed showed the opposite effect (Fig. 3F, G). Moreover, we measured SLC7A11 level by IHC in xenograft tumors, and found that the expression level of SLC7A11 in shMETTL3 group was significantly reduced compared with control group (Fig. 3H). Consistent with MeRIP-seq, MeRIP-qPCR analysis data showed that m^6^A SLC7A11 RNA was decreased upon METTL3 knockdown in both H1975 and PC9 cells, while overexpression substantially increased this (Fig. 3I, J), which indicated that the m^6^A modification of SLC7A11 directly affected its expression. To investigate whether the m^6^A modification affects the stability of SLC7A11 mRNA, we next performed RNA decay assays using the transcription inhibitor actinomycin D in different groups of cells at the indicated times. As the curves showed, METTL3 knockdown highly accelerated the half-life of SLC7A11 mRNA decay, while overexpression showed the opposite effect (Fig. 3K, L), which suggested its impact on SLC7A11 stability. In all, these results support the finding that SLC7A11 acts as a target of METTL3 in LUAD cells, which is dependent on its m^6^A methyltransferase activity.

### METTL3-mediated m^6^A modification of SLC7A11 promotes LUAD progression through inhibiting cell ferroptosis

**Fig. 4:**
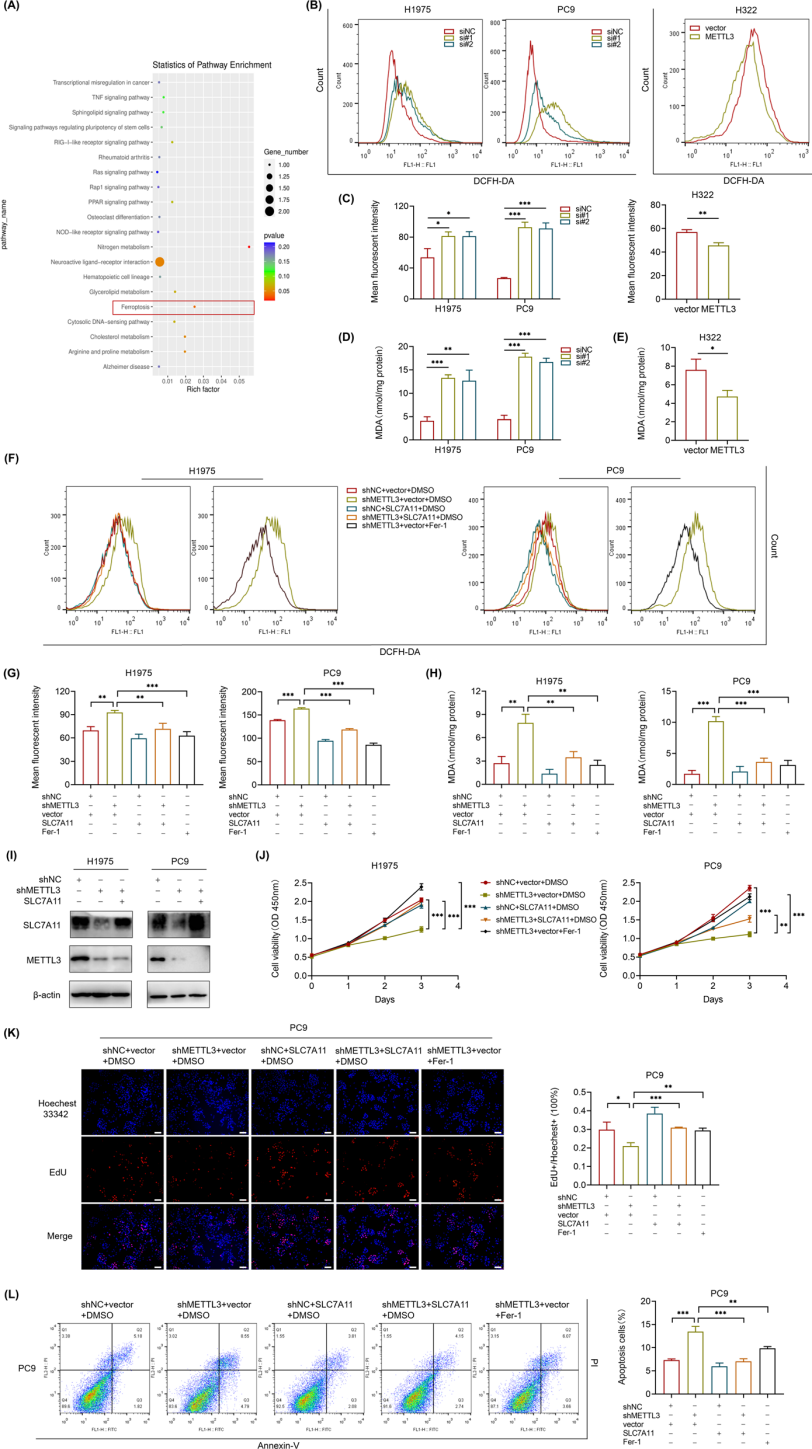
METTL3 inhibits LUAD ferroptosis through SLC7A11 m^6^A modification. **A** KEGG pathway enrichment analysis of MeRIP-seq data. **B** and **C** Flow cytometry analysis of intracellular ROS levels measured by DFCH-DA fluorescence with transfection of METTL3 siRNA or NC siRNA in H1975 and PC9 cells (48 h), and METTL3 overexpression in H322 cells (24 h). **D** and **E** Intracellular MDA levels normalized to corresponding protein contents in METTL3 knockdown or overexpression cells. **F**–**H**) Intracellular ROS and MDA levels in METTL3 stable knockdown H1975 and PC9 cells with SLC7A11 overexpression or Fer-1 treatment (1 μM) for 48 h. **I** The transfection efficiency of SLC7A11 overexpression in METTL3 stable knockdown H1975 and PC9 cells was confirmed by western blot. **J** CCK-8 assays showed the growth curves of H1975 and PC9 cells for 3 days after indicated treatments (Fer-1, 1 μM). **K** Representative images of EdU staining and the ratio of EdU positive cells to total Hoechest 33342 positive cells in METTL3 stable knockdown PC9 cells after SLC7A11 overexpression or Fer-1 treatment (1 μM) for 48 h, compared with control PC9 cells (Scale bar: 100 μm). **L** Flow cytometry analysis for apoptotic cell proportion (Q2 + Q3) by Annexin V-FITC/PI staining. *P < 0.05, **P < 0.01, ***P < 0.001

SLC7A11, a subunit of system Xc^−^, was recently shown to be overexpressed in multiple human cancers and to promote tumor progression partly by suppressing ferroptosis [25]. Intriguingly, the KEGG pathway enrichment of MeRIP-seq also revealed that ferroptosis was a significant pathway (Fig. 4A). Considering the prominence of ferroptosis among these pathways enriched by this MeRIP-seq, as well as the role of SLC7A11 in ferroptosis, we tried to explore the METTL3 regulation of SLC7A11 in ferroptosis of LUAD. ROS production and lipid peroxidation are critical mechanisms of ferroptosis-induced cell death [16]. Therefore, we measured intracellular ROS levels using DFCH-DA and the oxidative stress marker malondialdehyde (MDA). As expected, ROS accumulation and lipid peroxidation were much higher in METTL3 knockdown LUAD cells; likewise, METTL3 overexpression in H322 cells exhibited the opposite phenomenon (Fig. 4B–E). Furthermore, the elevated level in METTL3 stable knockdown cells could be completely attenuated by the presence of ferroptosis inhibitor ferrostatin-1 (Fer-1), or by SLC7A11 overexpression (Fig. 4F–H). The efficiency of SLC7A11 overexpression was confirmed by western blot (Fig. 4I). Moreover, CCK-8 assays showed that METTL3 knockdown could significantly inhibit H1975 and PC9 cell proliferation; however, this effect could be largely reversed by treatment with Fer-1 or of SLC7A11 overexpression (Fig. 4J). This was also verified by EdU assays in PC9 cells (Fig. 4K). Likewise, the promotion effect of cell apoptosis caused by METTL3 knockdown in PC9 cells was reduced with Fer-1 treatment or SLC7A11 overexpression as well (Fig. 4L). Taken together, these findings strongly demonstrate that METTL3 can inhibit LUAD cell ferroptosis via SLC7A11 m^6^A modification.

### METTL3 recruits YTHDF1 to regulate SLC7A11 mRNA stability and translation

**Fig. 5:**
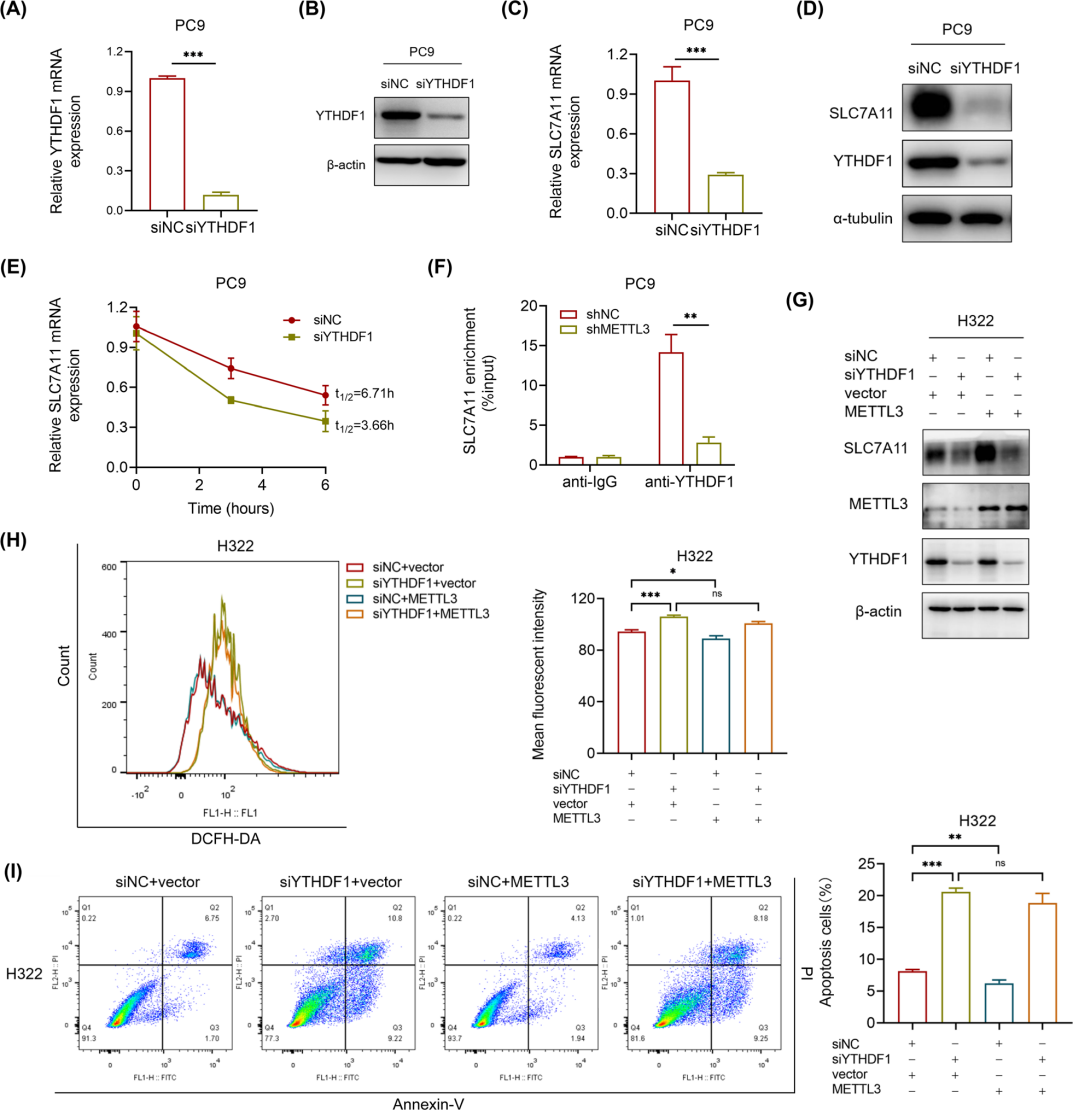
YTHDF1 is recruited by METTL3 to enhance SLC7A11 mRNA stability and translation. **A** and **B** The knockdown efficiency after 48 h transfection of YTHDF1 siRNA and negative control siRNA (siNC) in PC9 cells was confirmed by RT-qPCR and western blot. **C** and **D** The relative mRNA expression determined by RT-qPCR and the protein expression showed by western blot of SLC7A11 after YTHDF1 knockdown in PC9 cells. **E** SLC7A11 mRNA half-lives (t_1/2_) showed by RNA decay rates followed by RT-qPCR after YTHDF1 knockdown in PC9 cells. Data were collected at indicated timepoints (0 h, 3 h, and 6 h) with actinomycin D (Act D, 5 μg/mL) treatment. **F** RIP-qPCR revealed the binding enrichment of YTHDF1 to SLC7A11 in METTL3 stable knockdown and negative control PC9 cells. **G** The protein levels of SLC7A11 showed by western blot in YTHDF1 knockdown H322 cells with METTL3 overexpression, compared with control H322 cells. **H** Flow cytometry analysis of intracellular ROS levels in YTHDF1 knockdown H322 cells with METTL3 overexpression, compared with control H322 cells. **I** Flow cytometry analysis for apoptotic cell proportion (Q2 + Q3) by Annexin V-FITC/PI staining in H322 cells. *P < 0.05, **P < 0.01, ***P < 0.001, ns, not significant

The above results show that METTL3 regulates SLC7A11 expression to promote LUAD, yet the modification mechanism remains to be elucidated. YTHDF1 is recognized as one of the most important m^6^A readers responsible for mRNA translation [26]. Recent studies inspired us that METTL3 could recruit YTHDF1 to promote their target transcript stability [27, 28]. Thus, we knocked down YTHDF1 in PC9 cells by siRNA transfection, and the knockdown efficiency was validated by RT-qPCR and western blot (Fig. 5A, B). The downregulation of YTHDF1 markedly reduced the mRNA and protein expression of SLC7A11 in PC9 cells (Fig. 5C, D). Moreover, the half-life of SLC7A11 mRNA was also reduced in YTHDF1-downregulated PC9 cells (Fig. 5E), which indicated the essential role of YTHDF1 in regulating SLC7A11 mRNA stability. Furthermore, RIP-qPCR analysis showed that YTHDF1 bound directly to SLC7A11 mRNA, while METTL3 knockdown significantly reduced this binding efficiency (Fig. 5F). In addition, we found that the downregulated protein levels of SLC7A11 in YTHDF1-knockdown H322 cells could not be rescued by METTL3 overexpression (Fig. 5G), which implied that YTHDF1 was requisite for METTL3-mediated SLC7A11 m^6^A modification. Moreover, the ROS levels and the proportion of apoptosis cells were significantly increased when YTHDF1 was knocked down in H322 cells, while METTL3 overexpression had no discernible effect on these (Fig. 5H, I). In conclusion, our data reveal that YTHDF1 increase SLC7A11 expression through recognizing METTL3-mediated m^6^A-methylated SLC7A11 mRNA and enhancing its stability and translation.

### METTL3-mediated m^6^A modification of SLC7A11 is clinically related to LUAD progression

**Fig. 6:**
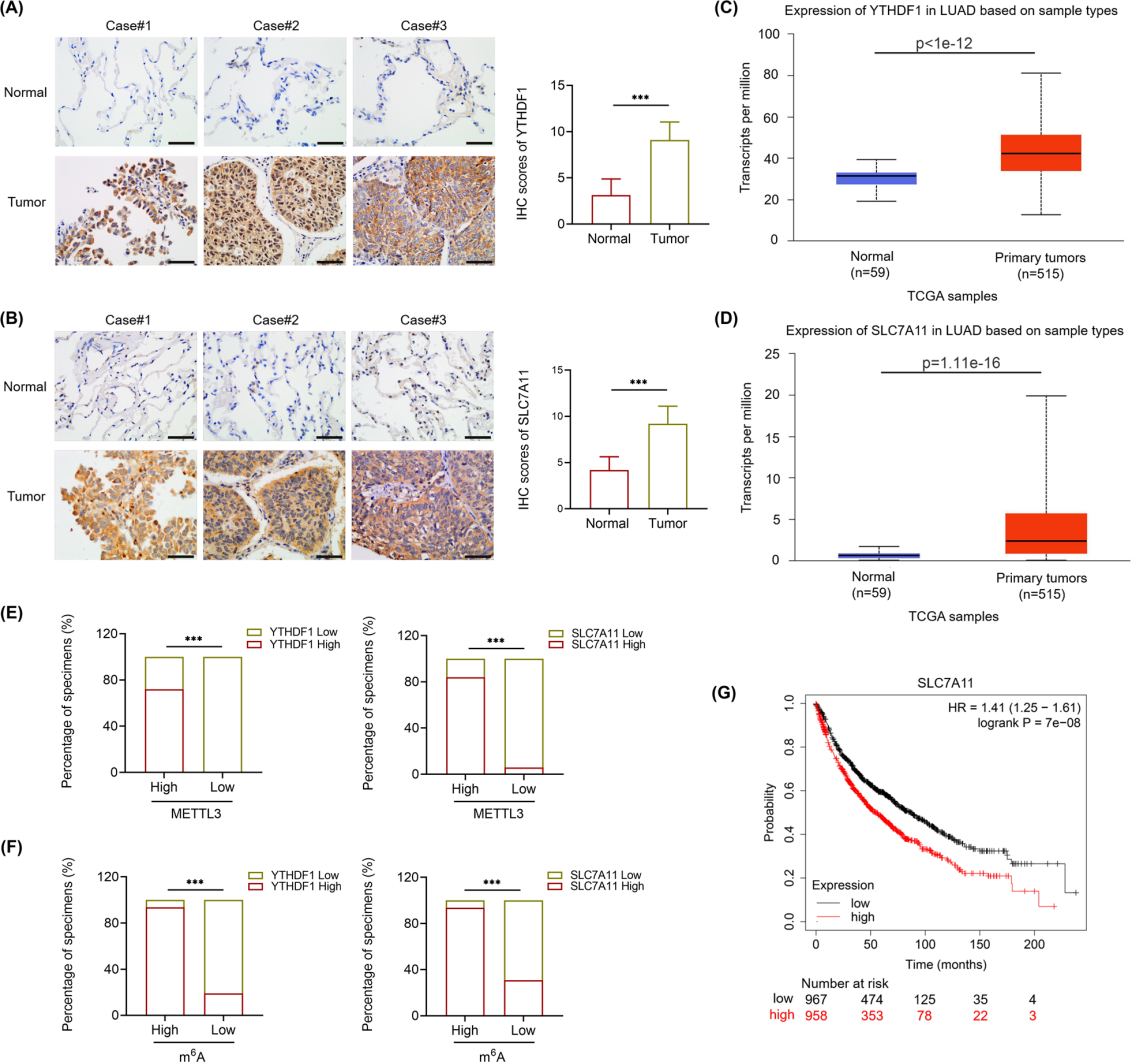
METTL3-mediated m^6^A modification of SLC7A11 is related to LUAD clinically. **A** and **B** Representative IHC images and IHC scores of YTHDF1 and SLC7A11 in 21 LUAD tissues and adjacent normal tissues (Scale bar: 50 μm). **C** and **D** Expression of YTHDF1 and SLC7A11 in LUAD patients based on TCGA samples using UALCAN platform. **E** The percentages of specimens with high or low METTL3 expression relative to the levels of YTHDF1 and SLC7A11. **F** The percentages of specimens with high or low m^6^A expression relative to the levels of YTHDF1 and SLC7A11. **G** Kaplan–Meier OS analysis of SLC7A11 expression in LUAD patients. (http://kmplot.com/analysis/)

To confirm the clinical significance of METTL3/SLC7A11 axis in LUAD, we next investigated the expression of YTHDF1 and SLC7A11 in LUAD human samples. As expected, both the expression of YTHDF1 and SLC7A11 were observably elevated in our LUAD samples by IHC and in the TCGA database using the UALCAN platform (Fig. 6A–D). What’s more, the expression of METTL3 and m^6^A were positively correlated with the expression of YTHDF1 and SLC7A11 in our LUAD clinical samples respectively (Fig. 6E, F). Moreover, LUAD patients with higher levels of SLC7A11 had poorer OS using the online analysis tool Kaplan–Meier Plotter (http://kmplot.com/analysis/) (Fig. 6G). Therefore, these data suggest that the METTL3-mediated m^6^A modification of SLC7A11 promotes human LUAD progression significantly.

## Discussion

In lung cancer, epigenetic alterations, such as DNA methylation, noncoding RNA expression, chromatin remodeling and posttranscriptional regulators, are key components in each step of tumor pathogenesis [29, 30]. Studies have showed that resistance to ferroptosis, the iron-dependent, nonapoptotic form of programmed cell death, is observed in many cell types and is closely related to the pathophysiological processes of many diseases, including neurodegeneration [31], liver fibrosis [32], ischemia/reperfusion-related damage [33–35] and various human cancers, as well as lung cancer [17]. During the past few years, accumulating evidence has revealed the essential role of the m^6^A modification in human cancers [9, 11]. Abnormal levels of m^6^A as well as its related proteins, including writers, erasers and readers, exhibit a strong correlation with tumor pathogenesis and progression. However, the m^6^A modification in LUAD still remains unclear. Recently, there have been several findings showing that m^6^A was closely linked with ferroptosis in cancers as well, which unveiled the great value of exploring the potential role of m^6^A in LUAD ferroptosis.

Our study showed elevated m^6^A levels in LUAD patients and cells, and METTL3 was the most significantly increased writer in LUAD compared with METTL14, WTAP, ZC3H13 or RBM15 in LUAD. Recent evidence has found that METTL3, depending on its methyltransferase activity, serves as an oncogene or tumor suppressor in different cancers [36]. For example, the depletion of METTL3 in acute myeloid leukemia (AML) cells induced cell differentiation and apoptosis through METTL3-mediated m^6^A modification on MYC, BCL2 and PTEN mRNA, thus delaying AML progression [37]. In colorectal cancer, METTL3 expression was found to be much higher in patients with higher FDG uptake, promoting cancer progression, which depends on cell glycolytic metabolism, by stabilizing HK2 and GLUT1 expression in an m^6^A-IGF2BP2/3-dependent manner [23]. In contrast, Jia et al. reported that ocular melanoma showed decreased m^6^A levels due to downregulation of METTL3 and demonstrated that METTL3-mediated m^6^A modification could promote the translation of tumor suppressor gene HINT2 [38]. Here, corresponded with previous researches in lung cancer [12, 39], our study demonstrated that METTL3 played an oncogenic role in LUAD tumorigenesis. Firstly, we conducted a series of loss-and gain-of-function assays in LUAD cells investigating the biological impact of METTL3. Results showed that METTL3 knockdown in H1975 and PC9 cells promoted cell proliferation and inhibited apoptosis, while overexpression in H322 cells exerted the opposite effect. Subsequently, METTL3 suppression in cell-derived xenografts exhibited a significant inhibitory effect in tumor growth, which further indicated the oncogenic role of METTL3 in LUAD tumorigenesis.

To further clarify the molecular mechanism of METTL3 in LUAD, we performed RNA-seq and MeRIP-seq analysis with stable METTL3 knockdown cells. Intriguingly, the KEGG analysis showed that ferroptosis was the closely correlated pathway. Meanwhile, SLC7A11, one reported regulator of ferroptosis was screened as the significant differently expressed gene affected by the level of METTL3. Our subsequent validation assays confirmed that METTL3 upregulated SLC7A11 mRNA methylation and enhanced its stability and translation, which was consistent with a previous study [40]. As is previously shown, SLC7A11 overexpression in cancer cells promotes ferroptosis resistance and thus influencing cancer growth, invasion, and metastasis and leads to an unfavorable prognosis [41]. Additionally, SLC7A11 was also found to be essential for tumor growth by relieving oxidative stress in some oncogenic KRAS-mutant cancers, including pancreatic ductal adenocarcinoma, colorectal adenocarcinoma and LUAD [42, 43].

Since iron-dependent ROS production and the accumulation of lipid peroxidation products are the main characteristics of ferroptosis [14, 44], we assessed intracellular ROS and lipid peroxidation levels to further explore the potential relation among METTL3, SLC7A11 and ferroptosis. We found that METTL3 knockdown significantly promoted LUAD cell ferroptosis, but further treated with ferroptosis inhibitor, ferrostatin-1, which could inversely inhibits lipid preoxidation [45], almost reversed the increase of LUAD cell ferroptosis and the decrease of LUAD cell proliferation caused by METTL3 deficiency. Additionally, we also demonstrated that SLC7A11 overexpression could partly rescue these effects in METTL3 knockdown LUAD cells, which represented that SLC7A11 acted as a target of METTL3 in LUAD in terms of both function and mechanism.

Recent studies have illustrated that m^6^A readers can recognize the m^6^A sites of mRNA transcripts and participate in multiple processes of RNA metabolism [46]. Among these, YTHDF1 tends to stabilize the transcript and promote mRNA translation, while several studies have demonstrated that METTL3 enhances targeted mRNA stability and translation in a YTHDF1-dependent manner in cervical cancer [27], oral squamous cell carcinoma [28] and liver cancer [47]. Besides, YTHDF1 was also found to be an oncogene in NSCLC, as it regulated the translational efficiency of CDK2, CDK4, and cyclin D1 [22]. As expected, our validation experiments confirmed that METTL3 promoted SLC7A11 mRNA stability and translation through YTHDF1 recruitment in LUAD cells. Moreover, the expression of YTHDF1 and SLC7A11 in LUAD tissues were positively correlated with METTL3 and m^6^A level, which indicated the clinical significance of METTL3-mediated m^6^A modification of SLC7A11 in LUAD progression.

## Conclusion

In summary, our work supports the oncogenic role of METTL3 in LUAD tumorigenesis and reveals its regulatory role in ferroptosis. Mechanistically, METTL3 promotes LUAD progression through SLC7A11 m^6^A modification in a YTHDF1-dependent manner. Moreover, SLC7A11 expression is correlated with poor prognosis of LUAD patients. Thus, targeting METTL3 and METTL3-mediated m^6^A modification of SLC7A11 might be promising diagnostic and therapeutic strategy for LUAD.

## Supplementary Information

**Additional file 1: Fig. S1.** (A-D) Expression of METTL14, WTAP, ZC3H13 and RBM15 in LUAD patients based on TCGA samples using UALCAN platform.

**Additional file 2: Table S1.** The sequence of primers for qPCR.

## Data Availability

The data that support the findings of this study are available on reasonable request from the corresponding author.

## Abbreviations

Fer-1: Ferrostatin-1
IHC: Immunochemistry
LUAD: Lung adenocarcinoma
m6A: N6-methyladenosine
MDA: Malondialdehyde
MeRIP: Methylated RNA immune-precipitation
METTL3: Methyltransferase-like 3
NSCLC: Non-small-cell lung cancer
RIP: RNA immune-precipitation
ROS: Reactive oxygen species
RT-qPCR: Real-time quantitative PCR
SLC7A11: Solute carrier 7A11
TCGA: The Cancer Genome Atlas
YTHDF: YT521-B homology domain family of proteins
