# Supplementary material for: METTL3 promotes lung adenocarcinoma tumor growth and inhibits ferroptosis by stabilizing SLC7A11 m6A modification
Source: Cancer Cell Int. 2022 Jan 7;22:11. doi: 10.1186/s12935-021-02433-6 (PMC8742440; doi:10.1186/s12935-021-02433-6)
Supplement: Supplementary file 2 — Additional file 2: Table S1. The sequence of primers for qPCR. [file 12935_2021_2433_MOESM2_ESM.pdf]

Table S1 The sequence of primers for qPCR

Primer	Sequence
β-actin-F	CATGTACGTTGCTATCCAGGC
β-actin-F	CTCCTTAATGTCACGCACGAT
METTL3-F	TTGTCTCCAACCTTCCGTAGT
METTL3-R	CCAGATCAGAGAGGTGGTGTAG
SLC7A11-F	CGTCCTTTCAAGGTGCCACT
SLC7A11-R	GGCAGATTGCCAAGATCTCAAG
YTHDF1-F	ACCTGTCCAGCTATTACCCG
YTHDF1-R	TGGTGAGGTATGGAATCGGAG
P2RY11-F	AGCTCCTATGTGCCCTACCA
P2RY11-R	GCGGCCATGTAGAGTAGAGG
LACC1-F	GTGTTGCTATGGCTACAGTGAA
LACC1-F	GTCCTACTGAAGGTCCAAGTACA
ZDBF2-F	CAAGGATATTGCAGTTATTGCCG
ZDBF2-R	AGACGATGACCCAGTATTCACAT
S1PR3-F	GGATGTGCTGGCTCATTGC
S1PR3-R	CAGGATGGTAGAGCAGTCAGG
CSF1-F	AGACCTCGTGCCAAATTACATT
CSF1-R	AGGTGTCTCATAGAAAGTTCGGA
TTL-F	CCTTCGTGGTACGCGATGAG
TTL-R	CAGCATCAGGTTGAATCTGGG
CA5B-F	TATGATCCCGGCTTAAAACCAC
CA5B-R	GGTAGTTGTGTTCCAGGGGTC
OSGIN1-F	CCCGGTCATCATTGTGGGTAA
OSGIN1-R	GCTTCGTGTAGGGTGTGTAGC
STING1-F	AGCATTACAACAACCTGCTACG
STING1-R	GTTGGGGTCAGCCATACTCAG
PDPK1-F	GGAACAGCGCAGTACGTTTCT
PDPK1-R	CTCGTTTCCAGCTCGGAATGG
ULBP2-F	AGCAACTGCGTGACATTCAG
ULBP2-R	GCCATCCTATACAGTCTCCCA
ZNF292-F	AGCTGTGCCAGACACTCCTA
ZNF292-R	CCAAGCGTTCCAGAACCAAG
EID3-F	GGCGAACAACTCCTTAACCGA
EID3-R	TCAGACCCACGAACAGAAACA
GALNT4-F	AAAAGTTGTGAGCCGAGCAG
GALNT4-R	CGAGCTCAGGTACTTCCCAG
APOL1-F	TGGACTACGGAAAGAAGTGGT
APOL1-R	CCTCCTTCAATTTGTCAAGGCTT

PIK3R2-F	TCACCTTCTGCTCCGTTGTG
PIK3R2-R	GGAGGTCCGTGTGTACTCTTC
ERCC5-F	CACCAAGCGCAGAAGAACATT
ERCC5-R	ACCACTCTCCTTGACTCTACCT
SLC15A4-F	TGGGAGCGATCCTGTCGTTA
SLC15A4-R	GGAAGACCACAAAAGCAAGGC
SYTL4-F	CAACACTACAGTGATCGGACC
SYTL4-R	ACAACCCCGACAAGTATTGGT
OLR1-F	TTGCCTGGGATTAGTAGTGACC
OLR1-R	GCTTGCTCTTGTGTTAGGAGGT
GPRC5B-F	CCTCCTCCCTCAGTACGTGTC
GPRC5B-R	AAGGCAAACGTCAGCCCAAA
MSMO1-F	TGCTTTGGTTGTGCAGTCATT
MSMO1-R	GGATGTGCATATTCAGCTTCCA
TNFAIP3-F	TCCTCAGGCTTTGTATTTGAGC
TNFAIP3-R	TGTGTATCGGTGCATGGTTTTA
PSMB8-F	GGTCCTACATTAGTGCCTTACGG
PSMB8-R	CGCAGATAGTACAGCCTGCATT
TRAFD1-F	TCTGGATTGCATCCCAACTCC
TRAFD1-R	GGGCTGTAATGTTCCTTTGGT
NEAT1-F	GACCTCTCACCTACCCACCT
NEAT1-R	CTTGTACCCTCCCAGCGTTT
